# Perinatal outcomes after selective third‐trimester ultrasound screening for small‐for‐gestational age: prospective cohort study nested within DESiGN randomized controlled trial

**DOI:** 10.1002/uog.29130

**Published:** 2024-11-25

**Authors:** C. Winsloe, J. Elhindi, M. C. Vieira, S. Relph, C. G. Arcus, K. Coxon, A. Briley, M. Johnson, L. M. Page, A. Shennan, N. Marlow, C. Lees, D. A. Lawlor, A. Khalil, J. Sandall, A. Copas, D. Pasupathy, on behalf of the DESiGN Trial Team

**Affiliations:** ^1^ Department of Women and Children's Health, School of Life Course and Population Sciences Faculty of Life Sciences and Medicine, King's College London London UK; ^2^ Centre for Pragmatic Global Health Trials Institute for Global Health, University College London London UK; ^3^ The Reproduction and Perinatal Centre, Faculty of Medicine and Health University of Sydney Sydney Australia; ^4^ Department of Obstetrics and Gynecology University of Campinas (UNICAMP), School of Medical Sciences São Paulo Brazil; ^5^ Women's Health Division Royal London Hospital, Barts Health NHS Trust London UK; ^6^ School of Nursing and Midwifery University of Central Lancashire Preston UK; ^7^ Caring Futures Institute, College of Nursing and Health Sciences Flinders University Adelaide Australia; ^8^ Department of Surgery and Cancer Imperial College London London UK; ^9^ West Middlesex University Hospital, Chelsea & Westminster Hospital NHS Foundation Trust London UK; ^10^ UCL Institute for Women's Health University College London London UK; ^11^ Department of Metabolism, Digestion and Reproduction Imperial College London London UK; ^12^ MRC Integrative Epidemiology Unit University of Bristol Bristol UK; ^13^ Population Health Science, Bristol Medical School University of Bristol Bristol UK; ^14^ Fetal Medicine Unit, St George's University Hospitals NHS Foundation Trust University of London London UK; ^15^ Vascular Biology Research Centre, Molecular and Clinical Sciences Research Institute St George's University of London London UK

**Keywords:** antenatal diagnosis, cerebral intraventricular hemorrhage, fetal death, fetal growth restriction, fetal weight, hypoxia–ischemia, brain, missed diagnosis, perinatal mortality, pregnancy complications, prenatal ultrasonography

## Abstract

**Objective:**

In screening for small‐for‐gestational age (SGA) using third‐trimester antenatal ultrasound, there are concerns about the low detection rates and potential for harm caused by both false‐negative and false‐positive screening results. Using a selective third‐trimester ultrasound screening program, this study aimed to investigate the incidence of adverse perinatal outcomes among cases with (i) false‐negative compared with true‐positive SGA diagnosis and (ii) false‐positive compared with true‐negative SGA diagnosis.

**Methods:**

This prospective cohort study was nested within the UK‐based DESiGN trial, a prospective multicenter cohort study of singleton pregnancies without antenatally detected fetal anomalies, born at > 24 + 0 to < 43 + 0 weeks' gestation. We included women recruited to the baseline period, or control arm, of the trial who were not exposed to the Growth Assessment Protocol intervention and whose birth outcomes were known. Stillbirth and major neonatal morbidity were the two primary outcomes. Minor neonatal morbidity was considered a secondary outcome. Suspected SGA was defined as an estimated fetal weight (EFW) < 10^th^ percentile, based on the Hadlock formula and fetal growth charts. Similarly, SGA at birth was defined as birth weight (BW) < 10^th^ percentile, based on UK population references. Maternal and pregnancy characteristics and perinatal outcomes were reported according to whether SGA was suspected antenatally or not. Unadjusted and adjusted logistic regression models were used to quantify the differences in adverse perinatal outcomes between the screening results (false negative *vs* true positive and false positive *vs* true negative).

**Results:**

In total, 165 321 pregnancies were included in the analysis. Fetuses with a false‐negative SGA screening result, compared to those with a true‐positive result, were at a significantly higher risk of stillbirth (adjusted odds ratio (aOR), 1.18 (95% CI, 1.07–1.31)), but at lower risk of major (aOR, 0.87 (95% CI, 0.83–0.91)) and minor (aOR, 0.56, (95% CI, 0.54–0.59)) neonatal morbidity. Compared with a true‐negative screening result, a false‐positive result was associated with a lower BW percentile (median, 18.1 (interquartile range (IQR), 13.3–26.9) *vs* 49.9 (IQR, 30.3–71.7)). A false‐positive result was also associated with a significantly increased risk of stillbirth (aOR, 2.24 (95% CI, 1.88–2.68)) and minor neonatal morbidity (aOR, 1.60 (95% CI, 1.51–1.71)), but not major neonatal morbidity (aOR, 1.04 (95% CI, 0.98–1.09)).

**Conclusions:**

In selective third‐trimester ultrasound screening for SGA, both false‐negative and false‐positive results were associated with a significantly higher risk of stillbirth, when compared with true‐positive and true‐negative results, respectively. Improved SGA detection is needed to address false‐negative results. It should be acknowledged that cases with a false‐positive SGA screening result also constitute a high‐risk population of small fetuses that warrant surveillance and timely birth. © 2024 The Author(s). *Ultrasound in Obstetrics & Gynecology* published by John Wiley & Sons Ltd on behalf of International Society of Ultrasound in Obstetrics and Gynecology.

## INTRODUCTION

The World Health Organization's (WHO's) Every Newborn Action Plan (ENAP) has called for an international drive to end preventable perinatal death by 2030. National strategies aimed at the mitigation of risk vary according to the predominant etiology of stillbirth, however, placental dysfunction is a common cause globally^1^, ^2^, ^3^. In high‐income countries, up to half of all stillbirths occur in fetuses that are small‐for‐gestational age (SGA), yet antenatal SGA detection is poor^4^. Fetal growth restriction carries an increased risk of stillbirth as well as short‐term adverse perinatal outcomes and long‐term health implications^5^, ^6^, ^7^.

The use of routine ultrasound in the third trimester of pregnancy for fetal growth surveillance and SGA detection has been associated with significantly improved detection of SGA, although over one‐third remain undetected^4^. Concerns with this approach have been raised over the implication that a false‐positive diagnosis (a fetal diagnosis of SGA for a neonate who is appropriate‐for‐gestational age (AGA)) could result in more (iatrogenic) preterm or early‐term births and increased morbidity, though this is dependent on the timing of third‐trimester ultrasound screening^8^, ^9^. There is inconclusive evidence from randomized controlled trials of a reduction in perinatal mortality or severe adverse neonatal outcome with the utilization of routine third‐trimester ultrasound screening^10^. Economic studies have not demonstrated universal ultrasound screening policies to be cost‐effective^11^. Therefore, in many high‐income settings, selective third‐trimester ultrasound programs are in place that recommend serial ultrasound surveillance only for women at a heightened risk of fetal growth anomaly. In low‐risk women, screening is based on routine uterine fundus palpation and measurement, with ultrasound being recommended if deviation of growth from expected norms is observed^12^. Within a selective screening program we have demonstrated previously that mothers who are at high risk of SGA (and who therefore qualify for serial ultrasound surveillance of fetal growth) are indeed more likely to have a SGA fetus detected before birth^13^. Conversely, undetected cases of SGA are more likely to occur in low‐risk women and in those who are overweight, yet these mothers represent approximately two‐thirds of the SGA population^13^. There is a paucity of information based on outcomes of screening classification for SGA in a selective ultrasound program.

The aim of this study was to investigate the incidence of adverse perinatal outcomes following selective third‐trimester ultrasound screening among cases with (i) false‐negative compared with true‐positive SGA diagnosis and (ii) false‐positive compared with true‐negative SGA diagnosis.

## METHODS

### Study design

This was a prospective cohort study using routinely collected electronic data from the DEtection of Small for Gestational age Neonate (DESiGN) trial. DESiGN was a prospective, multicenter, UK‐based, cluster randomized controlled trial conducted between November 2016 and March 2019 comparing the Growth Assessment Protocol (GAP) to standard care in the antenatal detection of SGA, in which no difference was found between randomized arms^14^. Details of the trial protocol, results and data management procedures have been published previously^14^, ^15^, ^16^. Ethical approval for the DESiGN trial was obtained through the Health Research Authority (HRA) Integrated Research Applications System (IRAS) from the London Bloomsbury Research Ethics Committee (Ref. 15/LO/1632), and the Confidentiality Advisory Group (Ref. 15/CAG/0195).

For this study, we included singletons born at > 24 + 0 and < 43 + 0 weeks' gestation, with no antenatal diagnosis of a congenital anomaly. Allocation of units to the intervention might have influenced ultrasound surveillance frequency and follow‐up, therefore, we included only the control (standard care) arm of the study and any patients included in the intervention arm before the implementation of GAP. Two sites in the intervention arm that were unable to provide ultrasound data during the preimplementation trial phase were excluded, as SGA detection status could not be determined for these babies. Pregnancies without a known perinatal outcome (live birth *vs* stillbirth) or gestational age (GA) at birth were also excluded. The study was designed and reported in accordance with strengthening the reporting of observational studies in epidemiology (STROBE) guidelines^17^.

### Outcomes

The primary perinatal outcomes explored were stillbirth (≥ 24 + 0 weeks' gestation^18^) and major neonatal morbidity. A diagnosis of stillbirth in the electronic patient record did not consistently distinguish between antepartum and intrapartum demise. The precise GA at diagnosis of stillbirth was not available in the dataset used. Neonatal morbidity was defined as major or minor based on definitions provided in the DESiGN primary trial^14^, ^15^. Major neonatal morbidity was a composite outcome, inclusive of any of the following: hypoxic ischemic encephalopathy, intraventricular hemorrhage, use of supplemental oxygen for > 28 days after birth, necrotizing enterocolitis, sepsis or retinopathy. We used the two primary outcomes to acknowledge the importance of avoiding a stillbirth without increasing major neonatal morbidity (e.g. through iatrogenic preterm birth) and reducing major neonatal morbidity without increasing the risk of stillbirth (e.g. by continuing a pregnancy post term). Minor neonatal morbidity, which included hypothermia, hypoglycemia or a requirement for nasogastric tube feeding, was explored as a secondary outcome.

### Exposure

Antenatally suspected SGA was defined as an ultrasound scan‐derived estimated fetal weight (EFW) below the 10^th^ percentile, based on the Hadlock fetal growth charts, at the last fetal growth scan (defined as any scan with fetal biometry conducted after 24 + 0 weeks' gestation) before birth^19^. The Hadlock formula is used commonly in the UK to estimate fetal weight *in utero* ^20^. SGA at birth was defined as a birth weight (BW) below the 10^th^ percentile, according to UK population references^21^. Non‐SGA was defined as an EFW or BW ≥ 10^th^ percentile. The SGA cut‐off definition was selected in line with UK guidance^12^, ^22^.

Using these definitions for SGA screening, four exposure categories were considered. A true positive was a fetus suspected to be SGA by EFW antenatally and confirmed to be SGA at birth. A false positive was a fetus suspected to be SGA by EFW but was non‐SGA at birth. A true negative was a fetus suspected to be non‐SGA by EFW and was indeed non‐SGA at birth. A false negative was a fetus suspected to be non‐SGA by EFW but was in fact SGA at birth.

### Management of missing data

Multiple imputation of missing data where appropriate has been described previously^16^. Imputed data are presented only as percentages to provide a meaningful result for the average values generated by the 10 imputed datasets.

### Statistical analysis

The statistical analysis was conducted using Stata/MP v17 (Stata Corp. LLC, College Station, TX, USA). The study population was divided into two groups. First, suspected SGA, comprising true positives and false positives. Second, suspected non‐SGA, including true negatives and false negatives. The maternal and neonatal characteristics are described according to antenatal SGA detection status. Demographic and pregnancy characteristics are described for each group with percentages (for multiply imputed data) and additionally with counts (*n*/*N*) for a sensitivity analysis using non‐imputed available‐case data or mean ± SD, as appropriate. Differences were assessed using chi‐square test or two‐sample *t*‐test, as appropriate.

The sample GA and BW percentiles of each of the true‐positive, false‐negative, true‐negative and false‐positive detection samples are described. Sample median (interquartile range (IQR)) is reported alongside frequency histograms to visualize the data.

To address the primary and secondary outcomes, two comparisons were devised. The first comparison was between false‐negative and true‐positive SGA diagnoses (BW < 10^th^ percentile by definition), to understand the implication of an antenatally unidentified SGA fetus. The second comparison investigated false‐positive *vs* true‐negative SGA diagnoses (BW > 10^th^ percentile), facilitating understanding of the implication of falsely identifying a fetus as SGA. Two comparisons, rather than one four‐way comparison, better reflected clinical concerns and ensured that adjustments for BW percentile were meaningful within each comparison.

For each comparison, unadjusted and adjusted regression models were used to assess differences in outcome associated with the exposure of interest. All primary and secondary outcomes were binary, therefore, all models were equipped with a logistic link function. The stillbirth outcome was adjusted for the following variables: maternal age, ethnicity, body mass index, index of socioeconomic deprivation, parity, smoking, chronic hypertension, diabetes mellitus, pre‐eclampsia, gestational hypertension, gestational diabetes mellitus and BW percentile, as well as the cluster site and DESiGN trial phase. Details of the data management of these variables have been reported previously^14^, ^16^. Stillbirth was not adjusted for GA at birth because the exact gestation of *in‐utero* fetal death was unknown and, although it may only differ minimally from the GA at birth, the GA at birth is determined commonly by iatrogenic procedures following a diagnosis of stillbirth. The major neonatal morbidity and minor neonatal morbidity outcomes were additionally adjusted for GA at birth because of the influence of premature birth on neonatal morbidity. A fractional polynomial model selection procedure determined that a square‐root‐transformed and squared‐transformed term for BW percentile, and a linear term for GA at birth, best fit all of the models. Results are reported as odds ratios (OR) with 95% CI.

## RESULTS

**Figure 1:**
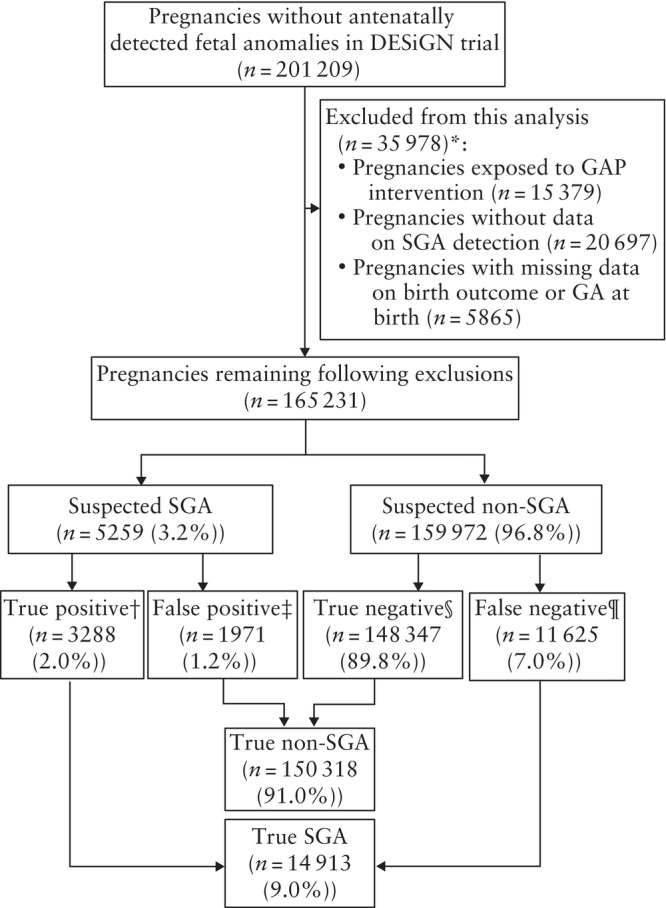
Flowchart summarizing study population of singleton pregnancies, included from DEtection of Small for Gestational age Neonate (DESiGN) trial^14^, that underwent selective third‐trimester ultrasound screening for small‐for‐gestational age (SGA). *Exclusion populations overlap. †SGA neonate that was correctly suspected as SGA antenatally (detected SGA). ‡Non‐SGA neonate that was incorrectly suspected as SGA antenatally. §Non‐SGA neonate that was correctly suspected as non‐SGA antenatally. ¶SGA neonate that was suspected as non‐SGA antenatally (undetected SGA). GA, gestational age; GAP, Growth Assessment Protocol.

Of the 201 209 singleton pregnancies without a congenital anomaly detected antenatally, born after 24 + 0 weeks' gestation and included in the DESiGN trial, we excluded pregnancies that had been exposed to the GAP intervention (*n* = 15 379), those without the data required for SGA screening classification (*n* = 20 697) and those without a known birth outcome or GA at birth (*n* = 5865). This resulted in a study population of 165 231 pregnancies, including 14 913 (9.0%) true SGA neonates. In this study population, 3.2% (*n* = 5259) of all fetuses were suspected antenatally to be SGA, and the SGA detection rate (the proportion of SGA neonates (*n* = 14 913) who were correctly detected (*n* = 3288) before birth) was 22.0%. The rates of SGA classification were as follows: 2.0% (*n* = 3288) were true positive, 1.2% (*n* = 1971) were false positive, 89.8% (*n* = 148 347) were true negative and 7.0% (*n* = 11 625) were false negative. The study population is presented in Figure 1.

The maternal, pregnancy and neonatal characteristics of the population are presented in Table 1. An antenatal suspicion of SGA (*vs* non‐SGA) was more likely when maternal age was < 20 years (3.7% *vs* 2.2%), 20–24 years (13.0% *vs* 10.2%) or ≥ 40 years (5.9% *vs* 5.3%), in Asian, black or multiethnic mothers (27.0% *vs* 18.8%, 14.8% *vs* 14.0%, 2.4% *vs* 2.0%, respectively), in the most deprived (24.2% *vs* 21.9%), in those who were underweight (7.1% *vs* 3.4%) or nulliparous (53.9% *vs* 47.9%), in those who smoked (9.6% *vs* 4.9%) as well as in those with gestational diabetes (4.6% *vs* 4.1%) and hypertensive disorders (chronic hypertension 2.2% *vs* 1.1%, gestational hypertension 2.2% *vs* 1.2%, pre‐eclampsia 4.2% *vs* 0.9%). A fetus suspected of being SGA antenatally was more likely to be born earlier (mean, 37.6 *vs* 39.4 weeks' gestation) as well as at a lower birth‐weight percentile (11.1^th^ *vs* 48.2^th^), compared with those suspected of being non‐SGA. The stillbirth rate was over four times higher in those fetuses suspected of being SGA (14.1 *vs* 3.1 per 1000 births) compared with those not suspected of being SGA antenatally. A sensitivity analysis with non‐imputed available case data replicated the trends observed (Table S1).

**Table 1:** Maternal, pregnancy and neonatal characteristics of study population, according to antenatal suspicion of small‐for‐gestational age (SGA) or non‐SGA

Characteristic	Suspected SGA (*n* = 5259)	Suspected non‐SGA (*n* = 159 972)
MA at 12 weeks' gestation (years)	31.1 ± 5.9	31.7 ± 5.5
< 20 years	3.7	2.2
20–24 years	13.0	10.2
25–34 years	57.2	59.0
35–39 years	20.2	23.2
≥ 40 years	5.9	5.3
Ethnicity		
Asian	27.0	18.8
Black	14.8	14.0
Multiethnic	2.4	2.0
White	47.0	56.1
Other	8.7	9.1
IMD quintile		
1 (least deprived)	12.1	12.9
2	12.7	12.9
3	20.1	20.5
4	31.0	31.8
5 (most deprived)	24.2	21.9
BMI (kg/m^2^)	24.7 ± 5.5	25.6 ± 5.4
< 18.5 kg/m^2^	7.1	3.4
18.5–24.9 kg/m^2^	53.4	50.4
25.0–29.9 kg/m^2^	24.7	28.3
30.0–34.9 kg/m^2^	9.6	11.9
35.0–39.9 kg/m^2^	3.5	4.1
≥ 40.0 kg/m^2^	1.8	1.9
Parity		
0	53.9	47.9
1	29.4	32.5
2	9.9	11.7
3	4.0	4.4
≥ 4	2.8	3.5
Smoker	9.6	4.9
Pre‐existing comorbidity		
Chronic hypertension	2.2	1.1
Pre‐existing diabetes	1.4	1.4
Pregnancy complication		
Gestational hypertension	2.2	1.2
Pre‐eclampsia	4.2	0.9
Gestational diabetes	4.6	4.1
Cephalic presentation at birth	91.7	95.9
GA at birth (weeks)	37.6 ± 3.0	39.4 ± 1.9
> 24 + 0 to 27 + 6 weeks	2.0	0.4
28 + 0 to 31 + 6 weeks	4.3	0.5
32 + 0 to 36 + 6 weeks	18.4	4.4
37 + 0 to 39 + 6 weeks	56.0	48.6
≥ 40 weeks	19.3	46.1
Birth‐weight percentile	11.1 ± 12.6	48.2 ± 26.7
Stillbirth rate (per 1000 births)	14.1	3.1

Data are given as mean ± SD or %, unless stated otherwise. Data using multiply imputed datasets provide only percentages of characteristics of interest. BMI, body mass index; GA, gestational age; IMD, index of socioeconomic deprivation; MA, maternal age.

### Consequences of false‐negative SGA diagnosis

**Figure 2:**
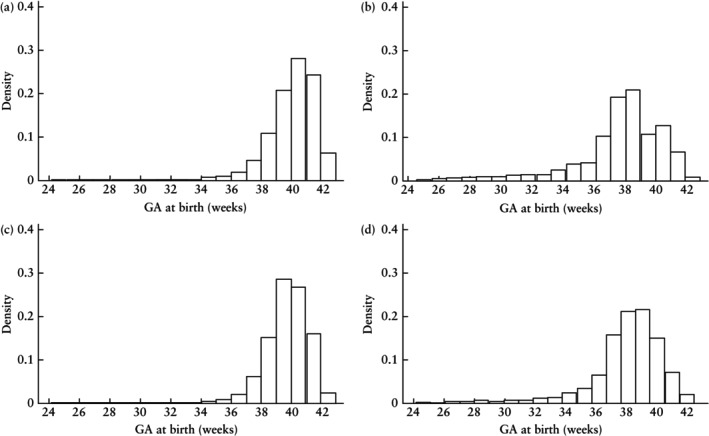
Histograms showing gestational age (GA) at birth, according to screening outcome of selective third‐trimester ultrasound examinations for small‐for‐gestational age: (a) false‐negative result (median GA at birth, 40.0 (interquartile range (IQR), 39.0–41.0) weeks); (b) true‐positive result (median GA at birth, 38.1 (IQR, 36.7–39.6) weeks); (c) true‐negative result (median GA at birth, 39.7 (IQR, 38.9–40.6) weeks); and (d) false‐positive result (median GA at birth, 38.3 (IQR, 37.0–39.4) weeks).

**Figure 3:**
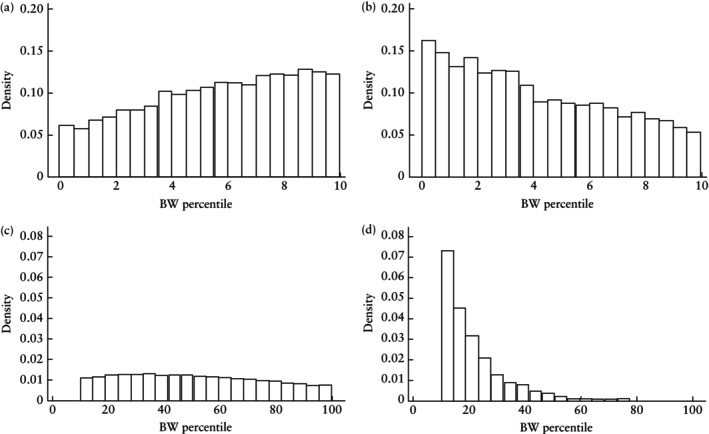
Histograms showing birth‐weight (BW) percentile, according to screening outcome of selective third‐trimester ultrasound examinations for small‐for‐gestational age: (a) false‐negative result (median BW percentile, 5.8 (interquartile range (IQR), 3.5–8.0)); (b) true‐positive result (median BW percentile, 3.6 (IQR, 1.7–6.4)); (c) true‐negative result (median BW percentile, 49.9 (IQR, 30.3–71.7)); and (d) false‐positive result (median BW percentile, 18.1 (IQR, 13.3–26.9)).

Undetected SGA babies (false negatives) were born almost 2 weeks later (median GA at birth, 40.0 (IQR, 39.0–41.0) weeks) than those who were detected as SGA (true positive) (median GA at birth, 38.1 (IQR, 36.7–39.6) weeks) (Figure 2). The SGA phenotype was less severe in false‐negative babies (median BW percentile, 5.8 (IQR, 3.5–8.0)) compared with true‐positive babies (median BW percentile, 3.6 (IQR, 1.7–6.4)) (Figure 3). Compared with true‐positive SGA cases, false negatives were associated with a significantly higher risk of stillbirth (adjusted OR (aOR), 1.18 (95% CI, 1.07–1.31)) and were significantly less likely to experience both major (aOR, 0.87 (95% CI, 0.83–0.91)) and minor (aOR, 0.56 (95% CI, 0.54–0.59)) neonatal morbidity (Table 2).

**Table 2:** Perinatal outcomes according to whether antenatal small‐for‐gestational age (SGA) was diagnosed correctly in SGA cohort

Outcome	False negative (missed SGA)	True positive (detected SGA)	Univariable analysis (OR (95% CI))	Multivariable analysis (aOR (95% CI))*
Stillbirth	13.5§	18.0§	0.73 (0.66–0.79)	1.18 (1.07–1.31)
Major neonatal morbidity†	7.1	16.2	0.40 (0.39–0.41)	0.87 (0.83–0.91)
Minor neonatal morbidity‡	5.2	19.1	0.23 (0.22–0.24)	0.56 (0.54–0.59)

Data are given as %, unless stated otherwise. Data using multiply imputed datasets provide only percentages of characteristics of interest.

* Adjusted for maternal age, ethnicity, body mass index, index of socioeconomic deprivation, parity, smoking, chronic hypertension, diabetes mellitus, pre‐eclampsia, gestational hypertension, gestational diabetes mellitus and birth‐weight percentile. Major and minor neonatal morbidity multivariable analysis adjusted additionally for gestational age at birth.

† Composite outcome inclusive of hypoxic ischemic encephalopathy, intraventricular hemorrhage, oxygen use for over 28 days after birth, necrotizing enterocolitis, sepsis or retinopathy.

‡ Composite outcome inclusive of hypothermia, hypoglycemia or requirement for nasogastric tube feeding.

§ Per 1000 births. aOR, adjusted odds ratio; OR, odds ratio.

### Consequences of false‐positive SGA diagnosis

Babies with a false‐positive SGA screening result were born approximately 1.5 weeks earlier than true‐negative cases (median GA at birth, 38.3 (IQR, 37.0–39.4) weeks *vs* 39.7 (IQR, 38.9–40.6) weeks) (Figure 2). Babies with a false‐positive result were smaller than those with a true‐negative result, with a lower median BW percentile (18.1 (IQR, 13.3–26.9) *vs* 49.9 (IQR, 30.3–71.7), respectively) (Figure 3). The risk of stillbirth (aOR, 2.24 (95% CI, 1.88–2.68)) and minor neonatal morbidity (aOR, 1.60 (95% CI, 1.51–1.71)) were increased significantly in the false‐positive group compared with the true‐negative group, whereas the risk of major neonatal morbidity was not (aOR, 1.04 (95% CI, 0.98–1.09)) (Table 3).

**Table 3:** Perinatal outcomes according to whether antenatal small‐for‐gestational age (SGA) was diagnosed correctly in non‐SGA cohort

Outcome	False positive (suspected SGA)	True negative (suspected non‐SGA)	Univariable analysis (OR (95% CI))	Multivariable analysis (aOR (95% CI))*
Stillbirth	7.6§	2.3§	3.45 (2.95–4.03)	2.24 (1.88–2.68)
Major neonatal morbidity†	11.1	5.6	2.11 (2.02–2.20)	1.04 (0.98–1.09)
Minor neonatal morbidity‡	10.2	2.8	3.90 (3.73–4.08)	1.60 (1.51–1.71)

Data are given as %, unless stated otherwise. Data using multiply imputed datasets provide only percentages of characteristics of interest.

* Adjusted for maternal age, ethnicity, body mass index, index of socioeconomic deprivation, parity, smoking, chronic hypertension, diabetes mellitus, pre‐eclampsia, gestational hypertension, gestational diabetes mellitus and birth‐weight percentile. Major and minor neonatal morbidity multivariable analysis adjusted additionally for gestational age at birth.

† Composite outcome inclusive of hypoxic ischemic encephalopathy, intraventricular hemorrhage, oxygen use for over 28 days after birth, necrotizing enterocolitis, sepsis or retinopathy.

‡ Composite outcome inclusive of hypothermia, hypoglycemia or requirement for nasogastric tube feeding.

§ Per 1000 births. aOR, adjusted odds ratio; OR, odds ratio.

## DISCUSSION

In this analysis of a large cohort that underwent selective third‐trimester ultrasound screening for SGA, we found that, when compared with true positives, a missed antenatal diagnosis of SGA (false negative) was associated with a significantly higher risk of stillbirth, but significantly less major and minor neonatal morbidity. We identified that a false‐positive screening result was also associated with a significantly increased risk of stillbirth, as well as minor neonatal morbidity, compared with a true‐negative screening result.

Compared with true‐positive cases, false‐negative cases were associated with less severe SGA. Nevertheless, these fetuses are at heightened risk of stillbirth. It may be that in missing the diagnosis of SGA, the pregnancy is not kept under close surveillance before birth and, for example, worsening growth restriction or placental dysfunction may then contribute to the stillbirth risk. Previous studies have observed a 2‐fold increase in the rate and risk of stillbirth when SGA remains undetected antenatally^5^, ^23^. Although this false‐negative group may represent the limitations of antenatal ultrasound in SGA detection, reduced growth velocity between the scan and birth resulting in an initially AGA fetus being SGA at birth is also plausible and may be associated with adverse perinatal outcome^24^. Our comparison using detected SGA (true positive) as a reference group describes only a modest increase in risk of stillbirth in undetected SGA (false negative) after adjustment, but the rate of stillbirth in this false‐negative group was still over 3‐fold greater than the current national average of 4.0 per 1000 births^25^. We found that, despite the higher adjusted odds of stillbirth observed in false‐negative cases (missed SGA diagnosis) compared with true positives, rates of major and minor neonatal morbidity were lower. These associations need to be interpreted within the context of the difference in GA distribution between false‐negative and true‐positive cases. The later GA at birth, perhaps due to less iatrogenic intervention, and higher BW percentiles observed in false‐negative cases contribute partly to the lower rate of neonatal morbidity. Adjustment for other unmeasured variables may help to explain the residual differences observed, such as method of conception, first‐trimester screening for dysfunctional placentation, uterine artery Doppler abnormalities, biomarkers and the interpretation of scan findings and subsequent management of the mode of birth, particularly in the context of SGA^26^, ^27^, ^28^. Environmental exposures and psychosocial factors may also play a role. Antenatal SGA screening, irrespective of a selective or universal ultrasound screening approach, will invariably be limited by the consequences of false‐positive results^9^, ^29^, ^30^. The potential for harm exists from these consequences, which include, but are not limited to, iatrogenic preterm birth^12^, ^31^. It is possible that the false‐positive group, which we found to be at a heightened risk of both stillbirth and minor neonatal morbidity compared with true negatives, captured fetuses that did not reach their growth potential. In other words, a non‐SGA fetus that experienced a reduction in growth velocity, and therefore was at increased risk of adverse perinatal outcome^6^, ^32^. False positives were below the 10^th^ percentile at the last scan (Table S2), but not at birth, yet were nonetheless smaller than true negatives; they may represent pregnancies affected by complications, for example, hypertension, diabetes, abnormal Doppler studies or placental abnormalities. Table 1 indeed suggests that those suspected to have SGA more commonly have comorbidities.

Improved antenatal detection of SGA, together with effective intervention, is needed to prevent stillbirth in small fetuses. Our previous work suggested that improving screening strategies in low‐risk women may be important, as they are more likely to have an undetected SGA baby^13^, ^30^. Alternative strategies that have been proposed include universal third‐trimester ultrasound screening. A UK‐based prospective cohort study suggested that the introduction of universal third‐trimester ultrasound screening (at 28 and 36 weeks' gestation) could increase the sensitivity of SGA detection from 20% (with selective sonography) to 57%. However, this was accompanied by an increase in the false‐positive rate from 2% to 10% and the study was limited to nulliparous women who have a higher incidence of SGA^4^. Randomized controlled trials in low‐risk pregnancies, attempting to determine the effect of universal third‐trimester ultrasound examinations, have demonstrated improvements in detection of SGA but without a reduction in severe adverse perinatal outcomes^10^. Our findings in this much larger cohort study highlight the limitations with respect to the primary outcome of interest in trials using composite measures incorporating both stillbirth and neonatal morbidity^4^, ^10^. We have demonstrated in this analysis that rates of stillbirth and those of major and minor neonatal morbidity behave differently in different subpopulations, highlighting the need for independent measures of outcome.

Alternative approaches using different estimated fetal weight percentile thresholds or as a continuum, combined with closer surveillance and timely birth, may reduce adverse perinatal outcome^33^, ^34^. A Swedish population‐based cohort study of over 200 000 singletons born at ≥ 37 weeks' gestation identified alternative threshold definitions for adverse outcomes associated with SGA that varied across different growth charts^34^. It is plausible that these thresholds would be different in preterm births. Evaluation of the use of different EFW thresholds or as a continuum merits further investigation, specifically with respect to the reduction of false‐negative and false‐positive cases to achieve greater balance of risk between stillbirth and neonatal morbidity.

### Strengths and limitations

This large cohort analysis enabled us to assess stillbirth and major neonatal morbidity independently. The overall stillbirth rate (3.4 per 1000 births) was reflective of nationally reported standards at the time^35^. Including both stillbirth and major neonatal morbidity as separate primary outcomes is a major strength of this study because it emphasizes the dual objectives of clinical management: to prevent stillbirth without inadvertently increasing major neonatal morbidity (e.g. due to iatrogenic preterm birth) and to minimize neonatal morbidity without raising the risk of stillbirth (e.g. by avoiding prolonged pregnancy), thereby providing a more comprehensive assessment of perinatal care strategies and their implications for both maternal and perinatal outcomes. Published studies looking at adverse perinatal outcomes often report stillbirth and perinatal morbidity as a composite measure^10^, ^36^, an approach that lacks the ability to decipher these mutually exclusive events. We also used a multiethnic cohort, with almost half of the participants belonging to minority ethnic groups. This supports our findings being potentially generalizable to other large multiethnic city populations.

Though SGA detection was poor in this multicenter study, it is a recognized limitation of selective antenatal ultrasound^4^, ^9^. The influence of different EFW and BW standards on SGA detection must be acknowledged. Our chosen standards are among the most widely used in the UK, allowing generalizability of our results to UK practice and similar populations. Although it has recently been demonstrated that paired EFW and BW charts may not always carry the highest sensitivity in screening for abnormalities of fetal growth^37^, we should recognize that a different combination of standards may improve sensitivity of SGA detection. Having birth outcomes with linked antenatal ultrasound data, meant that the antenatal diagnosis of SGA was based upon confirmed fetal biometry, rather than on documented clinical suspicion^9^, ^38^. Due to the nature of the electronic data available, we were unable to determine how the antenatal diagnoses, such as the detection of SGA or hypertensive disorders, influenced clinician decision‐making or patient perception of their choices once a diagnosis had been made, although an antenatal diagnosis of SGA did result in ultrasound scans performed closer to birth (Table S2). However, we observed that screen‐positive fetuses were born at a mean GA (38 weeks) reflective of national and international guidance^12^, ^31^. We were also unable to ascertain the cause of stillbirth or whether other factors contributed towards missed SGA cases, which are both important in future studies to achieve WHO's goal of eliminating preventable stillbirths^39^.

### Conclusions

In selective third‐trimester ultrasound screening for SGA, false‐negative and false‐positive screen results were associated with a significantly higher risk of stillbirth, when compared with true‐positive and true‐negative results, respectively. In order to address false‐negative results, SGA detection should be improved. However, it should be recognized that babies with a false‐positive SGA screening result are nonetheless a high‐risk population of small fetuses that warrant surveillance and timely birth.

## Supporting information


**Table S1** Available‐case sensitivity analysis, according to antenatal suspicion of small‐for‐gestational age (SGA) or non‐SGA
**Table S2** Characteristics of last ultrasound scan prior to birth, according to whether antenatal small‐for‐gestational age (SGA) was diagnosed correctly

## Data Availability

The data that support the findings of this study are available from the Confidentiality Advisory Group, Health Research Authority. Restrictions apply to the availability of these data, which were used under license for this study. Data are available from the authors with the permission of the Confidentiality Advisory Group, Health Research Authority.
