# Supplementary material for: Perinatal outcomes after selective third‐trimester ultrasound screening for small‐for‐gestational age: prospective cohort study nested within DESiGN randomized controlled trial
Source: Ultrasound Obstet Gynecol. 2024 Nov 25;65(1):30–8. doi: 10.1002/uog.29130 (PMC11693816; doi:10.1002/uog.29130)
Supplement: Supplementary file 1 — Table S1 Available‐case sensitivity analysis, according to antenatal suspicion of small‐for‐gestational age (SGA) or non‐SGA Table S2 Characteristics of last ultrasound scan prior to birth, according to whether antenatal small‐for‐gestational age (SGA) was diagnosed correctly [file UOG-65-30-s001.docx]

**Table S1** Available-case sensitivity analysis, according to antenatal suspicion of small-for-gestational age (SGA) or non-SGA

|  |  | SGA susp | Non-SGA susp |
| --- | --- | --- | --- |
|  |  | n/N (%) | n/N (%) |
| Age at 12 weeks’ (years) | <20 | 180/5,259 (3.4%) | 3,286/159,972 (2.1%) |
|  | 20-24 | 638/5,259 (12.1%) | 15,055/159,972 (9.4%) |
|  | 25-34 | 2,813/5,259 (53.5%) | 87,099/159,972 (54.4%) |
|  | 35-39 | 988/5,259 (18.8%) | 34,233/159,972 (21.4%) |
|  | ≥40 | 285/5,259 (5.4%) | 07,875/159,972 (4.9%) |
|  | Missing | 355/5,259 (6.8%) | 12,424/159,972 (7.8%) |
| Ethnicity | Asian | 1,361/5,259 (25.9%) | 28,472/159,972 (17.8%) |
|  | Black | 740/5,259 (14.1%) | 21,249/159,972 (13.3%) |
|  | Mixed | 119/5,259 (2.3%) | 3,049/159,972 (1.9%) |
|  | White | 2,328/5,259 (44.3%) | 84,450/159,972 (52.8%) |
|  | Other | 417/5,259 (7.9%) | 13,254/159,972 (8.3%) |
|  | Missing | 294/5,259 (5.6%) | 9,498/159,972 (5.9%) |
| IMD quintile | 1 (least deprived) | 626/5,259 (11.9%) | 20,451/159,972 (12.8%) |
|  | 2 | 661/5,259 (12.6%) | 20,468/159,972 (12.8%) |
|  | 3 | 1,045/5,259 (19.9%) | 32,545/159,972 (20.3%) |
|  | 4 | 1,611/5,259 (30.6%) | 50,382/159,972 (31.5%) |
|  | 5 (most deprived) | 1,264/5,259 (24.0%) | 34,649/159,972 (21.7%) |
|  | Missing | 52/5,259 (1.0%) | 1,477/159,972 (0.9%) |
| BMI (kg/m^2^) | <18.5 | 284/5,259 (5.4%) | 3,374/159,972 (2.1%) |
|  | 18.5-24.9 | 2,345/5,259 (44.6%) | 64,875/159,972 (40.6%) |
|  | 25.0-29.9 | 1,071/5,259 (20.4%) | 36,731/159,972 (23.0%) |
|  | 30.0-34.9 | 411/5,259 (7.8%) | 15,591/159,972 (9.7%) |
|  | 35.0-39.9 | 155/5,259 (2.9%) | 5,362/159,972 (3.4%) |
|  | ≥40.0 | 73/5259 (1.4%) | 2,386/159972 (1.5%) |
|  | Missing | 920/5,259 (17.5%) | 31,653/159,972 (19.8%) |
| Parity | 0 | 2,664/5,259 (50.7%) | 71,740/159,972 (44.8%) |
|  | 1 | 1,324/5,259 (25.2%) | 43,500/159,972 (27.2%) |
|  | 2 | 479/5,259 (9.1%) | 17,000/159,972 (10.6%) |
|  | 3 | 201/5,259 (3.8%) | 6,413/159,972 (4.0%) |
|  | ≥4 | 135/5,259 (2.6%) | 5,130/159,972 (3.2%) |
|  | Missing | 456/5,259 (8.7%) | 16,189/159,972 (10.1%) |
| Smoking | Yes | 502/5,259 (9.5%) | 7,794/159,972 (4.9%) |
| Pre-existing co-morbidities | Chronic Hypertension | 114/5,259 (2.2%) | 1,782/159,972 (1.1%) |
|  | Pre-Existing Diabetes | 074/5,259 (1.4%) | 2,152/159,972 (1.3%) |
| Pregnancy complications | Gestational hypertension | 113/5,259 (2.1%) | 1,868/159,972 (1.2%) |
|  | Pre-eclampsia | 216/5,259 (4.1%) | 1,370/159,972 (0.9%) |
|  | Gestational diabetes | 242/5,259 (4.6%) | 6,549/159,972 (4.1%) |
| Cephalic presentation |  | 4214/5,259 (80.1%) | 134,049/159,972 (83.8%) |
|  | Missing | 670/5,259 (12.7%) | 20,278/159,972 (12.7%) |
| Gestational age at birth (weeks) | >24+0 to 27+6 | 99/5,259 (1.9%) | 630/159,972 (0.4%) |
|  | 28+0 to 31+6 | 219/5,259 (4.2%) | 798/159,972 (0.5%) |
|  | 32+0 to 36+6 | 969/5,259 (18.4%) | 6,912/159,972 (4.3%) |
|  | 37+0 to 39+6 | 2,949/5,259 (56.1%) | 77,853/159,972 (48.7%) |
|  | ≥40 | 1,023/5,259 (19.5%) | 73,779/159,972 (46.1%) |
|  | Missing | n/a (excluded) | n/a (excluded) |
| Birth weight percentile |  | 5,259 | 159,972 |
|  | Missing | 0% | 0% |
| Birth outcome | Stillbirth | 74/5,259 (1.4%) | 481/159,972 (0.3%) |
|  | Livebirth | 5,185/5,259 (98.6%) | 159,491/159,972 (99.7%) |
|  | Missing | n/a (excluded) | n/a (excluded) |

SGA, small-for-gestational-age; IMD, index of socioeconomic deprivation; BMI, body mass index; SB, stillbirth.

**Table S2** Characteristics of last ultrasound scan prior to birth, according to whether antenatal small-for-gestational age (SGA) was diagnosed correctly

|  | TP | FN | TN | FP |
| --- | --- | --- | --- | --- |
| EFW percentile at last USS | 3.3  (1.0-6.4) | 25.3  (16.5-37.7) | 56.3  (37.6-75.8) | 6.6  (4.0-8.6) |
| GA at last USS (weeks’) | 36.6  (35.1-38.0) | 36.1  (34.9-37.7) | 36.0  (34.4-37.0) | 36.4  (35.1-37.9) |
| Last USS-birth interval (weeks’) | 1.0  (0.3-2.0) | 3.4  (1.7-5.3) | 3.1  (1.7-5.0) | 1.1  (0.4-2.3) |

*TP, true positive; FN, false negative; TN, true negative; FP, false positive; EFW, estimated fetal weight; USS, ultrasound scan; GA, gestational age*
